# Synergy in Polyphase Materials—Harnessing the Power of Glass and Ceramics

**DOI:** 10.3390/ma19030478

**Published:** 2026-01-25

**Authors:** Georgiy Shakhgildyan, Kai Xu, Michael I. Ojovan

**Affiliations:** 1 Department of Glass and Glass-Ceramics, Mendeleev University of Chemical Technology, 121165 Moscow, Russia; shakhgildian.g.i@muctr.ru; 2 State Key Laboratory of Silicate Materials for Architectures, Wuhan University of Technology, Wuhan 430030, China; kaixu@whut.edu.cn; 3 School of Chemical, Materials and Biological Engineering, The University of Sheffield, Sheffield S1 3JD, UK

Polyphase materials—where crystalline and vitreous constituents coexist and interact—offer a uniquely rich design space in which functionality can be engineered through phase assemblage, nanoscale morphology, interfaces, and defect chemistry [1,2,3,4,5,6,7]. The present Special Issue, ‘Synergy in Polyphase Materials: Harnessing the Power of Glass and Ceramics’, assembles eight open access papers that collectively demonstrate how targeted processing strategies translate into controllable microstructures and, ultimately, measurable performance gains across optical, thermal, electrical, tribological, and bioactive domains. A central theme in this Special Issue is the shift from viewing multiphase systems as an unavoidable complexity to treating them as a deliberate platform for property tuning. This is particularly clear in glass–ceramics, where phase selection, crystal size distribution, and the chemistry of residual glass decisively determines both their functional responses and reliability. The presence of vitreous components in polyphase materials is crucial due to their universality, which is provided by the amorphous state of this solid phase of amorphous materials [8]. They enable unlimited variations of compositions tolerating arbitrary ratios between components of glass which can be defined as, following Michael Faraday, “a solution of different substances one in another”. This is effectively used during the phase assemblage of composite materials, which is outlined in the contributions in this Special Issue, as well as in by many other dedicated topical collections such as [9,10]. Below, we briefly introduce the contributions to this Special Issue, highlighting their most important features.

Veselov et al. address a long-standing trade-off in transparent glass–ceramics: strengthening typically comes at the cost of optical scattering, while preserving transparency often limits mechanical performance [contribution 1]. By developing a sodium-modified ZnO–MgO–Al_2_O_3_–SiO_2_ (ZMAS) glass that remains fully compatible with two-stage crystallization into exclusively ZnAl_2_O_4_ (gahnite) nanocrystals, the authors enable an additional strengthening degree of freedom—chemical ion exchange in molten KNO_3_—without disrupting phase purity or optical transmittance. Importantly, the work underscores a broader, transferable concept: in transparent polyphase systems, mechanical robustness can be enhanced most effectively when bulk nanocrystallization and surface compressive-stress engineering are coupled rather than being treated as separate, potentially competing, interventions. This opens up new possibilities for electronic and photonic devices.

A complementary glass–ceramic contribution examines how redox conditions during melting influence phase transformations and defect-related optical responses in TiO_2_-nucleated lithium aluminosilicate (LAS) glass–ceramics. Maltsev et al. show that varying melting conditions (including the use of As_2_O_3_) change the kinetics of phase evolution and the appearance of optically active reduced titanium species (Ti^3+^ and Ti^3+^–Ti^4+^ pairs), while the overall transformation sequence remains largely preserved [contribution 2]. Beyond the specific LAS system, this study highlights an essential point for the design of polyphase materials: controlling redox and variable-valence species is not merely a “chemical detail”, but rather a decisive lever that couples directly to nucleation dynamics, intermediate amorphous-region chemistry, and the eventual functional signature of multiphase products.

The Special Issue also presents an instructive example of how unconventional thermal processing can be exploited to enhance optical performance in ceramic pigment systems. Monrós et al. explore delafossite cuprates CuMO_2_ (M = Mn, Fe, Cr) and doped/composite variants, aiming to approach ultra-black absorption metrics [contribution 3]. A key outcome is the demonstrated advantage of microwave-assisted firing in improving blackness compared to conventional electric kiln treatment, including cases where composite effects between the delafossite phase and associated chromium spinel become beneficial. The broader implication is methodological: for complex oxides where functional appearance sensitively depends on phase purity, defect populations, and microstructural uniformity, the heating modality itself can act as a “processing dopant”, accelerating reactions and shifting the balance between competing formation pathways.

Energy-related multiphase engineering is represented by the thermoelectric study of Thiem et al. on NbCoNi_x_Sn (*x* = 0–1), a system in which controlled introduction of Ni drives a transition from half-Heusler solid solutions at low *x* to half-Heusler/full-Heusler composites at higher *x* [contribution 4]. The work is valuable not only because it reports a composition window with an enhanced thermoelectric figure of merit (with the best performance around low Ni content), but also because it clarifies how interstitial occupation, secondary-phase emergence, and defect-driven phonon scattering co-evolve with electronic transport. In a field where incremental gains often depend on managing inherently multiphase microstructures, this paper contributes a coherent structure–property narrative that can guide rational optimization rather than purely empirical compositional screening.

The importance of enabling components—materials that do not constitute the primary functional phase, but determine real-world performance—appears in the tribology-focused contribution by Jianrong Liu et al., who developed a boron-modified phenolic resin for automotive friction materials [contribution 5]. By introducing B–O bonds and combining chemical modification with nano-alumina addition, the authors increased thermal decomposition resistance and report improvements in thermal fade behavior, supported by observations that are consistent with the formation of a more continuous friction film during braking. This study reinforces a practical message relevant to many polyphase systems: functional performance is frequently governed by interfacial integrity and the stability of transient, mechanically generated surface layers, making the chemistry of binders and secondary constituents as consequential as the selection of the “main” ceramic ingredients.

Two contributions address BaTiO_3_-derived perovskite ceramics, emphasizing how processing control shapes dielectric response through microstructural evolution. Feliksik et al. investigate Ba_0_._75_Ca_0_._25_TiO_3_ (BCT) ceramics and systematically connect the final sintering temperature to grain development, microstructural features, and dielectric behaviors [contribution 6]. Such processing–property coupling is central to perovskites because subtle shifts in density, grain-boundary characteristics, and defect distributions can dominate losses and stability, often more strongly than nominal composition alone. In a related but technologically distinct format, Zou et al. report Nb^5+^-doped BCZT-based ceramic thick films prepared via a scraping process and examine how sintering temperature influences the resulting microstructure and functional response in film form [contribution 7]. Together, these two studies articulate a shared conclusion: whether in bulk or thick-film architectures, reproducible dielectric/piezoelectric functionality requires process windows that are explicitly tuned to control microstructural heterogeneity rather than relying on composition as the sole “design variable”.

Finally, the bioactivity-oriented work by Qidong Liu et al. expands the scope of this Special Issue toward amorphous ceramics designed for controlled reactivity [contribution 8]. By modifying silicon oxycarbide (SiOC) with Ca or Mg and evaluating network depolymerization indicators alongside biological assays and simulated body fluid immersion, the study demonstrates that changes in network connectivity correlate with apatite formation behavior, while Ca- and Mg-modified compositions exhibit distinct response kinetics. This contribution underscores an important conceptual parallel with the other papers: in polyphase (or structurally complex) materials, performance is often dictated by how the “non-primary” structural motifs—here, network modifiers and non-bridging oxygen populations—govern interfacial reactions and time-dependent functional outcomes.

In summary, this Special Issue highlights the breadth and depth of polyphase design strategies for glass and ceramics. Across the contributions, recurring mechanisms (controlled crystallization, redox-mediated defect chemistry, processing-route-enabled phase selection, and interface/stress engineering) reappear in different material families and application contexts. The resulting picture is not one of fragmented subfields, but of a shared methodology: harnessing phase coexistence and interfacial phenomena as purposeful tools for property optimization. We anticipate that the insights provided here will stimulate further cross-fertilization between glass science, functional ceramics, and applied materials engineering, and also that they will support the development of next-generation polyphase materials where performance is not merely observed, but designed.
